# Vaginal birth after caesarean birth in Italy: variations among areas of residence and hospitals

**DOI:** 10.1186/s12884-018-2018-4

**Published:** 2018-09-24

**Authors:** Paola Colais, Katia Bontempi, Luigi Pinnarelli, Carlo Piscicelli, Ilenia Mappa, Danilo Fusco, Marina Davoli

**Affiliations:** 1 Department of Epidemiology, Lazio Regional Health Service, Via Cristoforo Colombo 112, 00142 Rome, Italy; 2 Cristo Re Hospital, Via delle Calasanziane, 25, 00167 Rome, Italy

**Keywords:** Caesarean section, VBAC, Health information systems, Hospital care

## Abstract

**Background:**

The rates of caesarean section (CS) are increasing globally. CS rates are one of the most frequently used indicators of health care quality. Vaginal Birth After Caesarean (VBAC) could be considered a reasonable and safe option for most women with a previous CS. Despite this fact, in some European countries, many women who had a previous CS will have a routine CS subsequently and VBAC rates are extremely variable across countries. VBAC use is inversely related to caesarean use. The objective of the present study was to analyze VBAC rates with respect to caesarean rates and the variations among areas of residence, hospitals and hospital ownership types in Italy.

**Methods:**

This study was based on information from the Hospital Information System (HIS). We collected data from all deliveries in Italy from January 1, 2010 to December 31, 2014 and we considered only deliveries with a previous caesarean section. Applying multivariate logistic regression analysis, the adjusted proportions of VBAC for each Local Health Units (LHU), each hospital and by hospital ownership types were calculated. Cross-classified logistic multilevel models were performed to analyze within geographic, hospitals and hospital ownership types variations.

**Results:**

We studied a total of 77,850 deliveries with a previous caesarean section in Italy between January 1, 2010 and December 31, 2014. The proportion of VBAC in Italy slightly increased in the last few years, from 5.8% in 2010 to 7.5% in 2014. Proportions of VBAC ranged from 0.29 to 50.05% in Italian LHUs. The LHUs with lower proportions of VBAC deliveries were characterized by higher values for primary caesarean deliveries. Private hospitals showed the lowest mean of crude VBAC proportions but the highest variation among hospitals, ranging from 0 to 47.1%.

**Conclusions:**

Hospital rates of caesarean section for women with at least one previous caesarean section vary widely, and only some of the variation can be explained by case-mix and hospital-level factors, suggesting that additional factors influence practices. Identifying disparities in VBAC may have important implications for health services planning and targeted efforts to reduce overall rates of caesarean deliveries.

**Electronic supplementary material:**

The online version of this article (10.1186/s12884-018-2018-4) contains supplementary material, which is available to authorized users.

## Background

The rates of caesarean section (CS) are increasing globally. CS rates rose throughout Europe between 2004 and 2010. In 2010, the Italian CS rate (38.0%) was among the highest in the world and only the Netherlands, Slovenia, Finland, Sweden, Iceland and Norway had rates below 20% [1]. The growth of Italian CS rates slowed down over the last few years, both for women who had not had a previous caesarean delivery (primary) [2] and for women who had undergone a previous caesarean delivery (repeated) [3].

Higher rates could be considered inappropriate, and maternal and neonatal benefits may no longer outweigh the costs and risks associated with this procedure [4]. Primary caesarean deliveries, which comprise two-thirds of the overall CS rate, are an important target for reduction because they lead to increased risk for a repeat caesarean delivery [5–7]. However, repeated CS following previous CS is a significant factor contributing to overall increased CS rates [8].

Furthermore, most women with a previous CS could have a Vaginal Birth After Caesarean (VBAC) [9] on the basis of randomized controlled trials that compared outcomes for women planning a repeated elective caesarean with women planning a vaginal birth [10]. There is a clear evidence of an association between VBAC and lower rates of maternal deaths or complications for mothers and babies [10]. Nevertheless, VBAC rates are extremely variable across European countries [1]. The variation in CS rates across Europe might reflect national, regional and individual clinicians’ attitudes to clinical decision-making and the use of caesarean section is not related to patient’s clinical risk factors and pregnancy characteristics, but it could be ascribed to differences in clinical practices [1, 11].

In general, VBAC use is inversely related to caesarean use such that higher VBAC rates are associated with lower caesarean rates. Previous studies showed that variation in hospital caesarean rates is not related to a patient’s clinical risk factors, but it could be ascribed to differences in practice patterns [12].

The Italian National Health System provides healthcare services through public and private hospitals that are reimbursed by different systems:

- “Public hospital” have a global budget with a fixed amount of money for health care spending;
- “Teaching hospitals” and “Classified hospitals” (hospitals owned by religious congregations) are partially reimbursed by the Diagnosis Related Groups (DRG) system and have a budget to cover the remaining health care spending;
- “Private hospitals” are totally paid by the DRG system.

The types of hospital ownership, such as whether it is a private or public hospital, teaching or nonteaching hospital, are associated with different CS and VBAC rates. In general, public hospitals and teaching hospitals have lower rates of caesarean delivery and higher VBAC rates in respect to private hospitals [13–16].

Patient factors and patient-specific clinical characteristics have been studied as risk factors for both caesarean delivery and VBAC. Maternal age, race/ethnicity, prematurity, pre-eclampsia, twins, more than one caesarean and previous vaginal birth are the most commonly studied [17, 18].

The objective of the present study was to analyze VBAC rates with respect to caesarean rates and the variations among areas of residence, hospitals and hospital ownership types in Italy.

## Methods

### Data sources

This study was based on information from the Hospital Information System (HIS). The HIS collects data on hospital discharges in Italy and contains patient demographic data (gender, age, place of residence), admission and discharge dates, discharge diagnoses and procedures (International Classification of Diseases, 9th Revision, Clinical Modification ICD-9-CM), and the regional code of the admitting facility.

Data were collected within the framework of the National Outcome Program, active from 2010 in the Italian Health System. The program measures more than 150 outcome indicators with the aim of comparing primary and hospital care in Italy [19]. The results provided by the National Outcome Program are updated every year and are publicly available [2].

### Study population

We collected data from all deliveries in Italy from January 1, 2010 to December 31, 2014. To identify deliveries from the Hospital Information System, three different sources of information were used: diagnosis-related group, procedure codes and diagnosis codes.

We considered only deliveries with a previous caesarean section. Previous caesarean deliveries were defined on the basis of the diagnosis code during hospitalization for delivery, or the diagnosis-related groups or diagnosis codes or procedure codes reported in hospitalizations occurred during the previous 5 years. Details and ICD-9-CM codes are reported in Additional file 1.

Moreover, we excluded the following from the analysis:

- all deliveries of mothers who were not residents of Italy;
- mothers under the age of 10 years or over the age of 55 years;
- hospital discharges with a diagnosis of abortion (diagnosis codes ICD-9-CM: 634–639);
- stillborn cases (diagnosis codes ICD-9-CM: 656.4, V27.1, V27.4, V27.7);
- admissions not remunerated by the National Health Service (e.g., admission remunerated by private insurance or by out-of-pocket fee).

We cannot exclude women < 37 weeks of gestation (potentially not eligible for VBAC) because the information about week of gestation is not completely available in the Italian Hospital Information System.

### Outcomes

The proportion of VBAC was calculated as the ratio of vaginal births to the total number of deliveries by women with a previous caesarean section. Vaginal deliveries were defined as deliveries without a caesarean section.

### Exposure

The results were calculated for each Local Health Unit (LHU) (a body delegated by the National Health System to provide health care to a specific area), and hospital and by hospital ownership types (public hospital, teaching hospitals, classified hospitals, and private hospitals).

### Comorbidities

Data on maternal and neonatal clinical factors, potentially associated with the outcome under study, were collected based on primary and secondary discharge diagnoses from the HIS; information was retrieved from the index hospital admission and all hospital admissions in the previous two years. Details and ICD-9-CM codes are reported in Additional file 1. Maternal age was classified as < 24, 25–28, 29–33, 34–38, and ≥ 39 years. We also considered as risk factors the mother’s citizenship, the number of previous caesarean sections (1, > 2) and previous vaginal deliveries.

Moreover, we evaluated the proportion of factors that usually contraindicate a VBAC (placenta praevia 0.7%, > = 2 previous CS 2.6%, podalic 2.3%). Considering the low proportion of these factors in the study population and the fact that sometimes a VBAC was performed in women with these factors, according to previous studies [20–22], we preferred to include them in the adjusted model in order to evaluate their impact on the likelihood of VBAC.

### Statistical analysis

The proportions of VBAC for each LHU, each hospital and by hospital ownership types were calculated. We used multivariate regression analysis to assess the effect of each exposure under study on the likelihood of a VBAC, adjusting for other factors (maternal age and comorbidities) that could affect the outcome under study. Among all factors potentially associated with the outcome under study, maternal age was considered an a priori risk factor; the others were selected using a stepwise bootstrap procedure to assign an importance rank of predictors in the logistic regression analysis. In this approach, the logistic regression with all predictors was run 100 times on random samples drawn with replacement from the original data set. Only the risk factors identified as at least 30 times as significant (*p* < 0.05) were included in the predictive model.

To estimate the adjusted proportion of VBAC, a multivariate logistic regression analysis with no intercept, including centered covariates, was applied. This model estimates the log odds of VBAC with respect to Local Health Units and hospitals.

Adjusted proportions were obtained for each level of exposure by back-transforming parameter estimates with the following formula [23]:

$$ \mathrm{Adj}\ \mathrm{proportion}={\left[\exp\ \left(\mathrm{estimate}\right)/\left(1+\exp\ \left(\mathrm{estimate}\right)\right)\right]}^{\ast }\ \mathrm{k} $$

where k is a correction coefficient introduced to consider the nonlinear nature of the logistic model (Additional file 2).

To estimate the adjusted OR of VBAC by hospital ownership types, a multivariate logistic regression analysis was applied.

Furthermore, maps of adjusted proportions of VBAC and primary caesarean deliveries were produced to compare each Italian Local Health Unit (ArcGIS 9.2 software). The classes used in the maps have been calculated applying the Jenks natural breaks optimization algorithm [24].

Finally, cross-classified logistic multilevel models were performed to analyze geographic, hospitals and hospital ownership type variations [25].

The variance components were expressed in terms of Median Odds Ratios (MORs) [26].

All analyses were undertaken using SAS Version 9.2.

The National Agency of Regional Health Services (Rome, Italy) gave approval for the study conducted within the broader scope of the National Outcome Program.

## Results

**Fig. 1:**
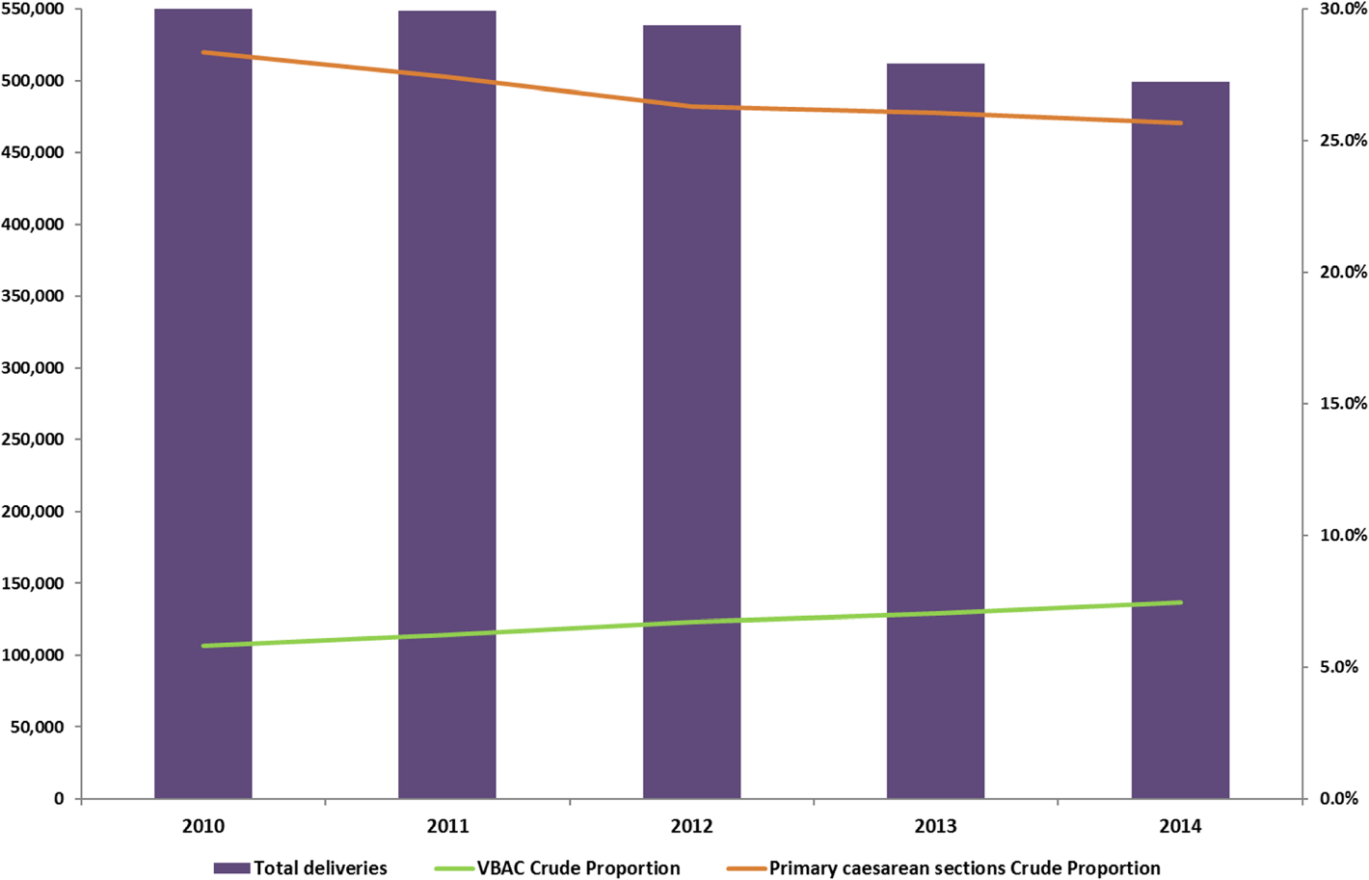
Crude proportion of VBAC deliveries, Italy 2010–2014

We studied a total of 416,758 deliveries with a previous caesarean section in Italy between January 1, 2010 and December 31, 2014. The proportion of VBAC in Italy slightly increased in the last few years, from 5.8% in 2010 to 7.5% in 2014; in the same period, the total number of deliveries and primary caesarean sections decreased (Fig. 1).

The risk factors included in the predictive model are reported in the Additional file 3. The likelihood of VBAC was lower in women aged 35–55 years than women aged 29–33 years, in Italian women, in women with more than 2 previous caesarean sections, eclampsia/pre-eclampsia, multiple pregnancy, malposition and malpresentation of the fetus, fetopelvic disproportion/excessive fetal growth affecting management of mother, intrauterine growth retardation and antepartum hemorrhage, abruptio placentae, and placenta previa and /or cord prolapse. On the other hand, the likelihood of VBAC was higher in women with previous vaginal deliveries, at-risk pregnancy, preterm labor, late pregnancy (over 40 weeks of gestation) and premature rupture of membranes. The strongest predictive factors for a VBAC were having a previous vaginal birth (OR = 8.76, *p* < 0.001) and late pregnancy (OR = 7.36, p < 0.001).

**Fig. 2:**
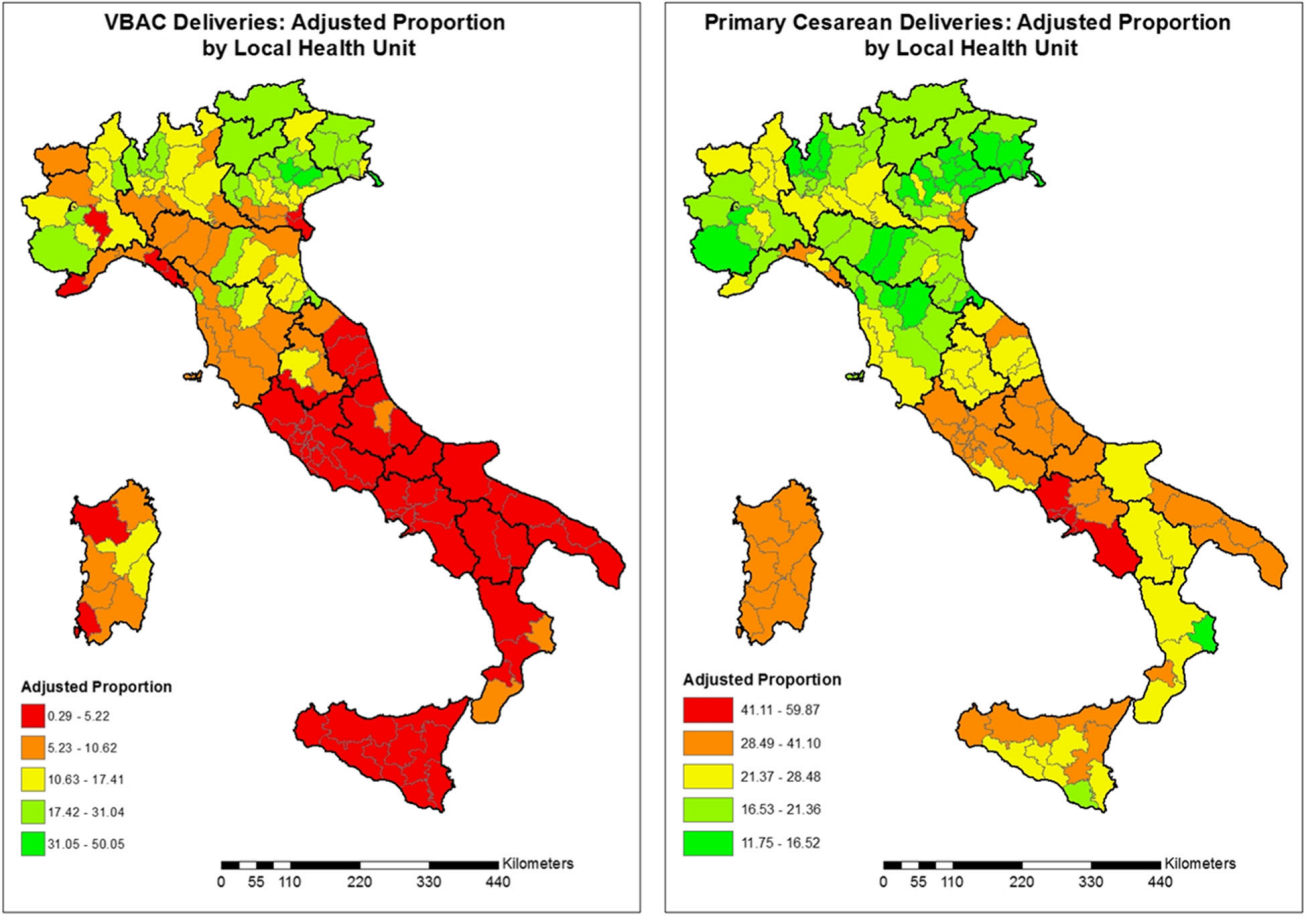
Adjusted proportion of VBAC and caesarean deliveries for Italian Local Health Units, 2014

Figure 2 shows the adjusted proportion of VBAC and primary caesarean deliveries for each Italian LHU in 2014. Comparing the maps, we noted that areas with a lower proportion of VBAC deliveries were characterized by higher values of primary caesarean deliveries.

The geographic variation of VBAC proportions ranged from 0.29 to 50.05% (MOR = 3.37) and was higher than the geographic variation of primary caesarean deliveries, ranging from 11.75 to 59.87% (MOR = 1.69).

**Fig. 3:**
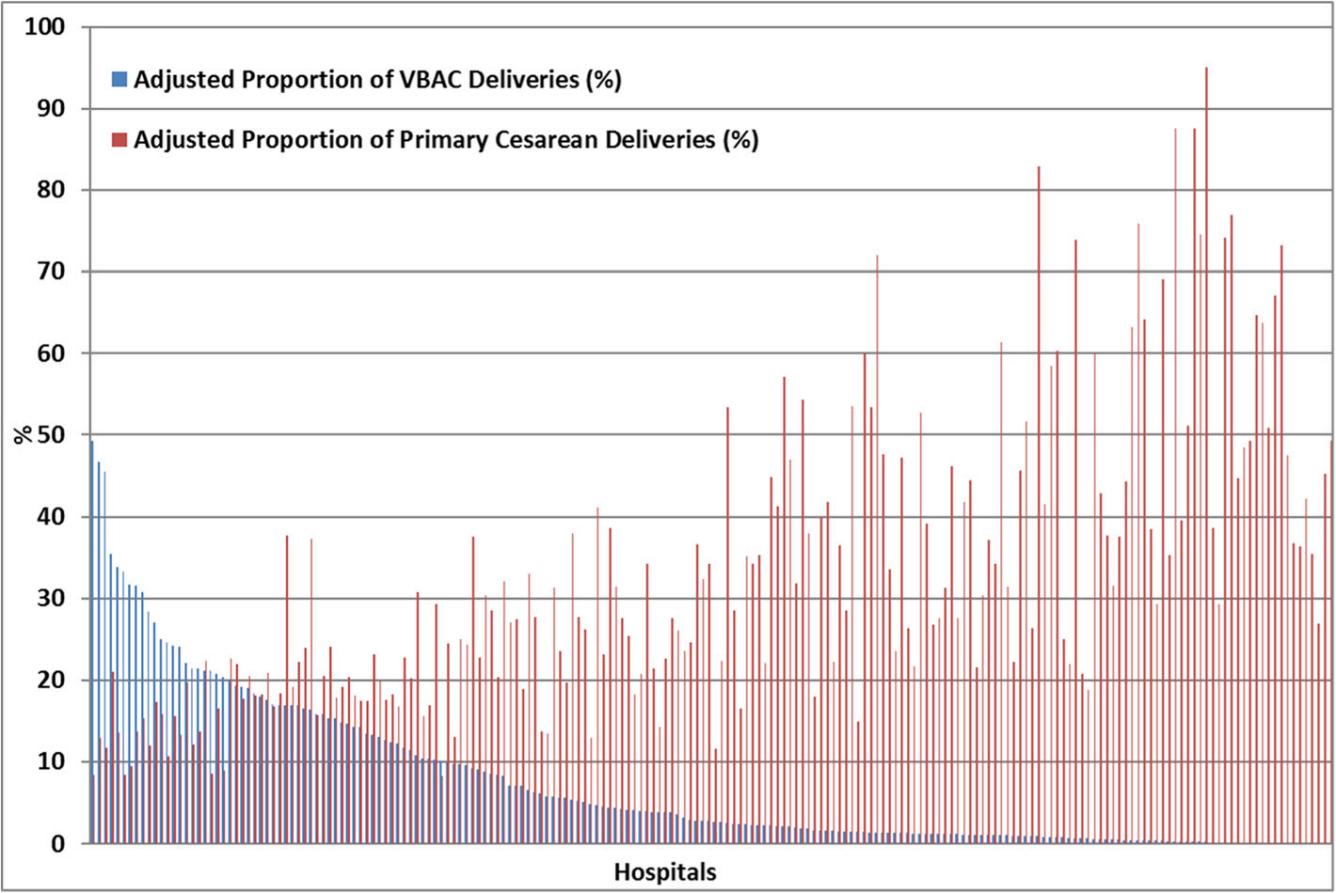
Adjusted proportion of caesarean and VBAC deliveries by hospital, 2014

A similar relationship between VBAC and primary caesarean deliveries could be found at the hospital level. Figure 3 shows that hospitals with a lower proportion of VBAC deliveries were characterized by higher values of primary caesarean deliveries. The VBAC proportion showed a higher variation between hospitals compared to primary caesarean deliveries (MOR = 7.01 vs MOR = 2.37, respectively).

Private hospitals showed the lowest crude VBAC proportion, whereas Public and Teaching hospitals showed the highest (Table 1). We observed a higher adjusted probability of VBAC in Public and Teaching hospitals (adjusted OR = 2.75 and adjusted OR = 2.75 respectively). However, the private category had a relevant variation between hospitals, ranging from 0 to 47.1% (MOR = 12.03), compared to the other types of hospital ownership.

**Table 1:** Adjusted OR of VBAC by hospital ownership types, 2014

Hospital ownership	n	Crude proportion			Crude OR	Adj OR	*p* value	Median OR
		%	% min	% max				
Public	49,353	8.69	0.00	41.86	3.35	2.75	<.0001	5.62
Teaching	10,332	8.68	0.89	37.75	3.34	2.76	<.0001	3.83
Classified	4382	5.48	0.68	20.30	2.04	1.87	<.0001	3.71
Private	13,783	2.76	0.00	47.12	1.00	1.00	–	12.03

## Discussion

The study evaluated VBAC rates with respect to caesarean rates and the variations among areas of residence, hospitals and hospital ownership types in Italy. We found that VBAC use is inversely related to caesarean use such that higher caesarean rates are associated with lower VBAC rates. There is clear evidence of this inversion relationship at the hospital level, but it can also be appreciated at the LHU level. Moreover, we found high geographic and hospital variations for both VBAC and primary caesarean deliveries. Although it was higher for VBAC, it was independent of the considered risk factors. Private hospitals showed the lowest mean of crude VBAC proportion with respect to Public and Teaching hospitals, but it had the highest variation among hospitals. These results are in line with previous studies [13–15, 27].

Our data suggested that younger women had higher VBAC success rates. Knight HE et al. found that women of non-white ethnicity and those who lived in deprived areas had a higher rate of attempted VBAC, but women of white ethnicity had a higher success rate [28]. We found that Italian women had a lower likelihood of success compared to women from Eastern Europe and developing countries citizenship but also compared to women coming from developed countries different from Italy. These results may reflect different preferences for modes of delivery or choice of hospital type for Italian women with respect to women from other countries.

Consistent with other studies and according to the Royal College of Obstetricians and Gynaecologists, we found that a prior vaginal delivery is the strongest predictive factor for a vaginal birth after caesarean [18, 29].

Although differences in the case mix may be important in explaining variation in hospital caesarean rates, these findings suggest that hospital planned repeat caesarean section rates vary markedly for reasons other than the individual’s characteristics.

Despite the existence of Italian national guidelines regarding the choice of caesarean section [30], our results suggest that its adoption is still very poor and heterogeneous and there is a need for interventions to change physicians’ attitudes and promote women’s empowerment. A wider implementation strategy for the existing clinical guidelines for the management of pregnant women should be promoted.

The main limitation of our study is the lack of information on personal patients’ data, i.e., height, body mass index, obstetric history and individual patient attitudes and physician decision-making processes.

However, this study is the first to compare VBAC rates respect to caesarean rates and the variations among areas of residence, hospitals and hospital ownership types in Italy in the recent past, using a large national dataset and routinely collected data on hospital admissions. Moreover, the hospital discharge data, even if have a value as a source for healthcare research, have several limitations that have been recognized [31]. On the other hand, this study uses a large data sample, a validated algorithm for the selection of patients and measures robust outcomes [31, 32].

## Conclusions

In Italy, VBAC use is inversely related to caesarean use such that higher caesarean rates are associated with lower VBAC rates at the hospital and LHU level. The geographical similarity between high CS and low VBAC rates is striking as is the wide range of rates for both. Hospital rates of caesarean sections for women with at least one previous caesarean section vary widely, and only some of the variation can be explained by case-mixes and hospital-level factors; other factors such as structural peculiarities and organizational and professional preferences seem to influence the use of caesarean section more strongly than women’s health conditions and pregnancy characteristics.

Identifying disparities in VBAC may have important implications for health services planning and targeted efforts to reduce overall rates of caesarean deliveries.

## Additional files

Additional file 1: List of codes used for selection of study population, outcome and comorbidities used for risk adjustment. The document contains the list of ICD-9-CM codes used for selection of study population, outcome and comorbidities used for risk adjustment (DOCX 19 kb)

Additional file 2: Correction coefficient. The document contains the formula of correction coefficient (k) used to consider the nonlinear nature of the logistic model. (DOCX 14 kb)

Additional file 3: Comorbidities included in a model to predict VBAC. The document contains the list of comorbidities included in the regression model and the associated Crude OR, adjusted OR and *p*-value. (DOCX 16 kb)

## Abbreviations

CS: Caesarean Section
DRG: Diagnosis Related Groups
HIS: Hospital Information System
ICD-9-CM: International Classification of Diseases, 9th Revision, Clinical Modification
LHU: Local Health Units
MOR: Median Odds Ratio
OR: Odds Ratio
VBAC: Vaginal Birth After Caesarean
