# Supplementary material for: Vaginal birth after caesarean birth in Italy: variations among areas of residence and hospitals
Source: BMC Pregnancy Childbirth. 2018 Sep 24;18:383. doi: 10.1186/s12884-018-2018-4 (PMC6154898; doi:10.1186/s12884-018-2018-4)
Supplement: Supplementary file 1 — List of codes used for selection of study population, outcome and comorbidities used for risk adjustment. The document contains the list of ICD-9-CM codes used for selection of study population, outcome and comorbidities used for risk adjustment (DOCX 19 kb) [file 12884_2018_2018_MOESM1_ESM.docx]

**Additional file 1: List of codes used for selection of study population, outcome and comorbidities used for risk adjustment**

**Codes used to identify deliveries:**

- **diagnosis-related group (DRG) 370–375**

**OR**

- **procedure codes ICD-9-CM: 72.x, 73.2, 73.5, 73.6, 73.8, 73.9, 74.0, 74.1, 74.2, 74.4, 74.99**

**OR**

- **diagnosis codes ICD-9-CM: V27.xx, 640.xy - 676.xy, where y = 1 or y = 2.**

**Codes used to identify previous caesarean section:**

- **during hospitalization for delivery**
  - **diagnosis code ICD-9-CM 654.2**
- **in hospitalizations occurred during the previous 5 years**
  - **diagnosis-related group (DRG) 370–371**

**OR**

- - **diagnosis codes ICD-9-CM 654.2, 669.7, V30.01, V31.01, V32.01, V33.01, V34.01, V36.01, V37.01, V39.01**

**OR**

- - **procedure codes ICD-9-CM 74.0, 74.1, 74.2, 74.4, 74.99.**

**Codes used to define caesarean section:**

- **diagnosis-related group (DRG) 370–371**

**OR**

- **diagnosis code ICD-9-CM 669.7, V30.01, V31.01, V32.01, V33.01, V34.01, V36.01, V37.01, V39.01**

**OR**

- **procedure codes ICD-9-CM 74.0, 74.1, 74.2, 74.4, 74.99.**

**Codes used to define comorbidities**

| **Risk factor** | **ICD-9-CM code** | |
| --- | --- | --- |
|  | **During hospitalization for**  **childbirth** | **During previous**  **hospitalizations** |
| Cancer | 140.0–208.9 | 140.0–208.9 |
| Anemias | 280-284, 285 (except 285.1), 648.2  (except 648.22, 648.24) | 280-284, 285 (except 285.1) |
| Coagulation defects | 286 | 286 |
| Heart diseases | 390-398, 410-429 | 390-398, 410-429 |
| Cardiovascular diseases in pregnancy | 648.5, 648.6 |  |
| Congenital anomalies of heart and circulatory system | 745-747 | 745-747 |
| Cerebrovascular disease | 433, 437, 438 | 430-432, 433, 434, 436, 437, 438 |
| Nephritis, nephritic syndrome and nephrosis | 580-589 | 580-589 |
| Unspecified renal disease in pregnancy, without mention of hypertension | 646.2 |  |
| Diffuse diseases of connective tissue | 710 | 710 |
| HIV | 042, 079.53, V08 | 042, 079.53, V08 |
| Disorders of thyroid gland | 240-246, 648.1 | 240-246 |
| Diabetes | 250.0-250.9, 648.0 | 250.0-250.9 |
| Hypertension | 401-405,642.0-642.3, 642.9 | 401-405 |
| Pre-eclampsia / eclampsia | 642.4-642.7 |  |
| COPD | 491-492, 494, 496 | 491-492, 494, 496 |
| Asthma | 493 | 493 |
| Cystic fibrosis | 277.0 | 277.0 |
| Acute pulmonary diseases | 480-487, 510-514 |  |
| Chronic pulmonary diseases | 500-508, 515-517 | 500-508, 515-517 |
| Tuberculosis | 010-018, 647.3 | 010-018 |
| Genital herpes | 054.1 |  |
| Other sexually transmitted diseases | 077.98, 078.88, 079.88, 079.98, 090- 099,  647.0- 647.2 |  |
| Antepartum hemorrhage, abruptio placentae/placenta previa and/or Cord prolapse | 641, 663.0 |  |
| Preterm labor | 644.1, 644.2 |  |
| Late pregnancy | 645 |  |
| Liver disorders in pregnancy | 646.7 |  |
| Polyhydramnios, oligohydramnios /  infection of the amniotic cavity | 657, 658.0, 658.4 |  |
| Premature rupture of membranes | 658.1 |  |
| Malposition and malpresentation of fetus | 652 (except 652.0, 652.1, 652.5) |  |
| Disproportion/Excessive fetal growth affecting management of mother | 653, 656.60, 656.61, 656.63 |  |
| Fetal abnormality | 655 |  |
| Intrauterine growth retardation | 656.5, 764 |  |
| Fetal distress | 656.3, 768 |  |
| Multiple pregnancy | 651, V27.2 –V27.9, V31-V37, 761.5 |  |
| Rh isoimmunization | 656.1 |  |
| Maternal conditions affecting fetus or newborn | 760.0, 760.1, 760.3 |  |
| Pregnancy at risk | 640, 644.0, V23.0, V23.2, V23.4, V23.5, V23.7, V23.8 |  |
| Assisted fertilization | V26 |  |
