# Supplementary material for: Vaginal birth after caesarean birth in Italy: variations among areas of residence and hospitals
Source: BMC Pregnancy Childbirth. 2018 Sep 24;18:383. doi: 10.1186/s12884-018-2018-4 (PMC6154898; doi:10.1186/s12884-018-2018-4)
Supplement: Supplementary file 2 — Correction coefficient. The document contains the formula of correction coefficient (k) used to consider the nonlinear nature of the logistic model. (DOCX 14 kb) [file 12884_2018_2018_MOESM2_ESM.docx]

**Additional file 2 - correction coefficient**

K is calculated as follows:

K= actual number of events/ $\sum_{j=1}^{m} p_{j}*n_{j}$

where pj are the adjusted proportions, n_j_ is the group size, and m is the number of groups.
