# Supplementary material for: Vaginal birth after caesarean birth in Italy: variations among areas of residence and hospitals
Source: BMC Pregnancy Childbirth. 2018 Sep 24;18:383. doi: 10.1186/s12884-018-2018-4 (PMC6154898; doi:10.1186/s12884-018-2018-4)
Supplement: Supplementary file 3 — Comorbidities included in a model to predict VBAC. The document contains the list of comorbidities included in the regression model and the associated Crude OR, adjusted OR and p-value. (DOCX 16 kb) [file 12884_2018_2018_MOESM3_ESM.docx]

**Additional file 3 – Comorbidities included in a model to predict VBAC.**

| **Condition** | **n** | **Crude OR** | **Adj OR** | **p value** |
| --- | --- | --- | --- | --- |
| 29-33 years | 23951 | 1.00 | 1.00 | - |
| 10-24 years vs 29-33 years | 3742 | 0.97 | 0.91 | 0.164 |
| 25-28 years vs 29-33 years | 9453 | 1.03 | 0.94 | 0.167 |
| 34-38 years vs 29-33 years | 28123 | 0.91 | 0.95 | 0.140 |
| 39-55 years vs 29-33 years | 12581 | 0.75 | 0.79 | 0.000 |
| Citizenship: Italian | 64677 | 1.00 | 1.00 | - |
| Citizenship: missing | 660 | 6.33 | 5.77 | 0.000 |
| Citizenship: Developed countries vs Italian | 351 | 2.39 | 2.31 | 0.000 |
| Citizenship: Eastern Europe vs Italian | 3742 | 1.94 | 1.81 | 0.000 |
| Citizenship: Developing countries vs Italian | 8420 | 2.31 | 2.12 | 0.000 |
| Number of previous caesarean sections: 1 | 75823 | 1.00 | 1.00 | - |
| Number of previous caesarean sections: >=2 | 2027 | 0.26 | 0.24 | 0.000 |
| Previous Vaginal Deliveries | 2552 | 8.74 | 8.76 | 0.000 |
| Pregnancy at risk | 338 | 1.57 | 1.73 | 0.003 |
| Eclampsia/pre-eclampsia | 762 | 0.51 | 0.48 | 0.000 |
| Preterm labor | 1243 | 1.81 | 1.82 | 0.000 |
| Late pregnancy | 403 | 8.64 | 7.36 | 0.000 |
| Multiple pregnancy | 981 | 0.24 | 0.24 | 0.000 |
| Malposition and malpresentation of fetus | 2164 | 0.14 | 0.10 | 0.000 |
| Fetopelvic disproportion/excessive development of the infant | 352 | 0.83 | 0.60 | 0.031 |
| Intrauterine growth retardation | 1081 | 0.56 | 0.56 | 0.000 |
| Premature rupture of membranes | 3584 | 3.57 | 3.53 | 0.000 |
| Antepartum hemorrhage, abruptio placentae, and placenta previa and/or Cord prolapse | 1002 | 0.21 | 0.18 | 0.000 |
